# Factors Associated with Dietary Diversity Score among Individuals with Type 2 Diabetes Mellitus

**Published:** 2014-12

**Authors:** Kee Fong Tiew, Yoke Mun Chan, Munn Sann Lye, Seng Cheong Loke

**Affiliations:** ^1^Department of Nutrition and Dietetics, Faculty of Medicine and Health Sciences, Universiti Putra Malaysia, 43400 UPM, Serdang, Malaysia; ^2^Institute of Gerontology, Faculty of Medicine and Health Sciences, Universiti Putra Malaysia, 43400 UPM, Serdang, Malaysia; ^3^Department of Community Health, Faculty of Medicine and Health Sciences, Universiti Putra Malaysia, 43400 UPM, Serdang, Malaysia; ^4^Department of Medicine, Faculty of Medicine and Health Sciences, Universiti Putra Malaysia, 43400 UPM, Serdang, Malaysia

**Keywords:** Cross-sectional studies, Dietary diversity, Diet quality, Type 2 diabetes mellitus, Malaysia

## Abstract

Studies on diet quality among individuals with type 2 diabetes mellitus (T2DM) are scarce. This cross-sectional study aimed to assess the diet quality and to determine its associated factors among individuals with T2DM at the Medical Outpatients Department, Serdang Hospital, Selangor, Malaysia, from July 2010 to March 2011. Subjects were interviewed for sociodemographic data. Diabetes history was retrieved from the hospital's *e*-database. Usual dietary intake was measured using a food frequency questionnaire, from which a dietary diversity score was obtained with two measures: Food Group Score and Serving Score were constructed based on the Malaysian Dietary Guidelines. Food Group Score was computed from the number of food groups consumed from five major food groups (grains, vegetables, fruits, meat, and dairy products) whereas Serving Score was computed from the number of servings consumed from the various food groups. Anthropometric measures, including weight, height, waist- and hip-circumference were examined. For data analyses, descriptive statistics, simple and multiple linear regression were conducted using IBM SPSS Statistics 20.0. A total of 113 subjects (50.4% female), with mean±SD age of 54.05±10.30 years and duration of diabetes of 11.25±9.05 years were studied. The mean Food Group Score and Serving Score were 4.12±0.79 and 12.75±3.50 respectively. Slightly more than one-third of the subjects achieved five food groups a day while less than 2% consumed a desirable number of servings from all food groups. Among the five food groups, dairy, and fruits were the least-frequently consumed foods. Lower education, lower personal income, working, non-insulin, overweight and obese subjects had significantly lower Food Group Score than their counterparts [F (6,106)=4.924, p<0.0001] whereas lower education, lower waist-to-hip ratio, overweight and obese subjects had significantly lower Serving Score than their counterparts [F (4,108)=7.520, p<0.0001]. There was a high proportion of individuals with T2DM, who failed to adhere to the national dietary guidelines. The importance of taking a well-balanced diet in accordance with the guidelines should be emphasized, especially among those with lower educational level through a simple and easy-to-understand approach.

## INTRODUCTION

Diabetes mellitus remains a major cause of mortality and morbidity worldwide. Factors associated with the continuous upward trend include population growth, ageing, urbanization, increasing prevalence of obesity as well as physical inactivity (1). The prevalence of diabetes worldwide is projected to increase from 8.3% in 2011 to 9.9% in 2030 (2). Among 80 most-populated countries in the world, Malaysia appears to have the highest prevalence of diabetes in the Western Pacific region (3). International Diabetes Federation predicted that the prevalence of diabetes in Malaysia is projected to reach 13.3% in 2030 (3). However, the worrying fact is that the prevalence of diabetes in Malaysia increases even faster than the projection, which recorded 11.6% in 2006 (4) and 15.2% in 2011 (5). It was ranked in the top 10 total burden of disease in Malaysia in terms of premature mortality (6). Individuals with diabetes are at higher risk of suffering from diabetes complications as it frequently co-exists with a constellation of cardiovascular (CVD) risk factors and metabolic syndrome (7,8).

Dietary management is crucial for preventing diabetes, managing existing condition, and preventing the development of diabetes complications (9). Assessing diet quality among individuals with diabetes may be beneficial for the development of diabetes management intervention, particularly for secondary and tertiary prevention to reduce the burden of disease. Diet quality was found to have a protective effect towards health outcomes, including a reduction of 17-42% for all-cause mortality, 18-53% for CVD mortality, and 14-28% for CVD risk (10). Dietary diversity score (DDS), as one of the diet quality indicator (11,12), was found to be inversely associated with CVD risk (13) and metabolic syndrome (14). Although dietary management is crucial for individuals with diabetes, the studies on diet quality among them are scarce (10,15). Type 2 diabetes mellitus (T2DM) is the most common type of diabetes, and it accounted for 90-95% of all diabetes cases (8). Therefore, this study aims to assess the diet quality as measured using DDS and to determine the factors associated with DDS among individuals with T2DM.

## MATERIALS AND METHODS

### Subjects

This cross-sectional study was conducted among 113 Malaysian men and women who were aged above 18 years, diagnosed with T2DM, with poor glycaemic control as defined by HbA1c ≥8.0%, and attended the Medical Outpatient Department of Serdang Hospital, a government-funded multispecialty hospital located in the district of Sepang in the state of Selangor, Malaysia, during July 2010-March 2011. The exclusion criteria included those who were severely impaired in vision, hearing, or speech and who were unable to communicate in English, Malay, Mandarin, or Cantonese. In addition, pregnant or lactating women and individuals who were diagnosed with cardiac failure, severe renal disease, gastrointestinal diseases, mental disorientation or other chronic medical conditions that required specific dietary restriction were excluded.

The sample-size was calculated based on Torheim and colleagues’ study (12) who found that about 22% of the variance for DDS was explained by sociodemographic characteristics. The effect-size was obtained using the formula as follows (16):


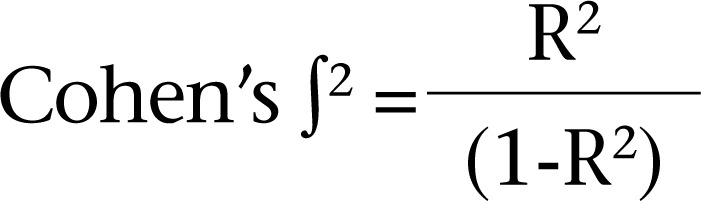


where R^2^ is the expected coefficient of determination.

The minimum sample-size comprised 92 subjects based on G*Power version 3.1.7 (Franz Faul, University of Kiel, Kiel, Germany) (17), with an expected medium effect-size of 0.282, power of 0.90, alpha (α) value of 0.05 for a multiple linear regression model with 13 predictors. The final sample-size was further increased to 108 after considering an estimated 15% non-response rate.

### Ethical clearance

Ethical approval for the study was obtained from the Medical Research and Ethics Committee of the Faculty of Medicine and Health Sciences, Universiti Putra Malaysia and the Ministry of Health Malaysia. The nature of the study was explained, and an informed consent was given by individuals to participate in the study.

### Instruments

All subjects were interviewed using a structured questionnaire pre-tested with face validity and content clarity. Diabetes history of subjects was retrieved through the hospital's electronic medical record database. Anthropometric measures, including weight, height, waist-circumference (WC), and hip-circumference were recorded using standardized procedures. Body mass index (BMI) was calculated as weight in kg divided by height in metre squared. The BMI classification was based on the WHO criteria (2004) (18), specifically for the Asian population as follows: BMI <18.5 kg/m^2^ underweight; 18.5-22.9 kg/m^2^ normal range; 23.0-27.4 kg/m^2^ overweight; 27.5-32.4 kg/m^2^ pre-obese; 32.5-37.4 kg/m^2^ obese Class I; and ≥37.5 kg/m^2^ obese Class II. Waist-to-hip ratio (WHR) was calculated as WC in cm divided by hip-circumference in cm. The classifications of WC and WHR were based on the WHO/IASO/IOTF criteria (2000) (19) and WHO criteria (1998) (20) respectively.

Usual dietary intake of subjects was assessed using a food frequency questionnaire which consisted of 28 food groups that were commonly consumed in Malaysia. Subjects were asked to recall the frequency and portion-size of foods and beverages that they consumed over the past month on a daily, weekly or monthly basis with the aid of household measurement tools. The reported frequency was converted to daily intake while the reported portion-size was converted to number of servings based on the serving-size recommended by the Malaysian Dietary Guidelines (MDG) (21) to generate DDS for subjects. Measures of DDS, namely Food Group Score (FGS) and Serving Score (SS) were modified from Kant *et al*. (22). The FGS reflected the number of food groups consumed daily from a total of five food groups—grains (cereals, tubers, and grains), fruits, vegetables, meat (fish, poultry, meat, eggs, and legumes), and dairy (milk and dairy products). The minimum amount to be credited as consumed for each food group was at least one-half serving per day based on the serving-size recommended in the MDG (21) (Table 1). One point was given for each food group consumed daily and added up to a maximum of five if all food groups were consumed daily. Meanwhile, the SS reflected the presence of achieving the minimum recommended number of servings for the five food groups—four servings daily for grains and two servings daily each of fruits, vegetables, meat, and dairy. Subjects who consumed below the minimum amount, which was less than one-half serving, were treated as having zero serving; intakes above the minimum amount but below the recommended serving-size were credited with one serving; intakes which were 1.5 times of the recommended serving-size were credited with 1.5 servings, and so on. One or two point(s) were awarded for the consumption of each serving of grains and other food groups respectively. The maximum score for each food group was four points while the maximum score for total SS was 20 points. Perfect score of 5 for FGS indicated consumption of all the 5 food groups daily whereas perfect score of 20 for SS indicated that the individuals were taking at least the minimum number of servings from all food groups as recommended in the MDG (21).

### Statistical analyses

The IBM SPSS Statistics 20.0 (SPSS Inc., Chicago, IL, USA) was used in all statistical analyses. Univariate analysis was conducted to describe the data. A series of simple linear regression models were applied. Variables that had p<0.25 in the simple linear regression models were chosen for backward stepwise multiple linear regression analysis to further examine the factors associated with DDS among the subjects. Statistical significance was indicated by p<0.05.

## RESULTS

A total of 113 subjects (50.4% female), with mean age of 54.05±10.30 years, comprising various ethnic groups (46.0% Malay, 28.3% Chinese, 23.0% Indian, and 2.7% other ethnicities) participated in the present study (Table 2). Majority of the study subjects were married (84.1%). Approximately one-third of the subjects had attained primary education (32.7%); more than one-third (37.2%) had monthly personal income of less than 500 MYR (Malaysian Ringgit) (US$ 158), and almost half (46.9%) had monthly household income of less than 3,000 MYR (US$ 950). On average, the subjects were diagnosed with T2DM for 11.25±9.05 years, and more than half (57.5%) were on insulin regimen. Most were overweight, pre-obese or obese (93.8%), and more than two-thirds (74.3-87.6%) were found to have abdominal obesity.

Table 3 presents the distribution of subjects by DDS. The mean FGS and SS were 4.12±0.79 and 12.75±3.50 points respectively. Slightly more than one-third of the subjects (34.5%) scored a perfect point of 5 for FGS while only a handful (1.8%) scored a perfect point of 20 for SS. Grains group was consumed daily by all subjects; however, only about half (54.0%) of them consumed four servings of cereals and grains. This was followed by meat (97.3%) and vegetable (95.6%) consumption. It is worth noting that only 38.9% and 51.3% of the subjects took at least 2 servings daily each of the meat and vegetable groups respectively. Although approximately three-quarters of the subjects (76.1%) consumed fruits daily, less than one-third of the subjects (29.2%) met the recommended number of servings of fruits, which were 2 servings per day. Dairy products were the least-frequently consumed foods. Less than half of the subjects (43.4%) consumed milk and dairy products on a daily basis, with a majority (89.4%) failing to achieve two servings of milk and dairy products.

**Table 1.** Number of servings according to food groups recommended by the Malaysian Dietary Guidelines (21)

Food group	Recommended number of servings/day*
Cereals, tubers, and grains†	4-8
Fruits‡	2
Vegetables	3
Fish, poultry, meat, eggs, and legumes	
Meat/poultry/egg¶ Fish¶ Legumes§	½−2 1 ½−1
Milk and dairy products^§^	1-3

*Based on 1,500-2,500 kcal/day, with calories from fat and sugars included;

^†^Based on 30 g carbohydrate per serving;

^‡^Based on 15 g carbohydrate per serving;

^¶^Based on 14 g protein per serving;

^§^Based on 7 g protein per serving

**Table 2.** Sociodemographics, diabetes history, and anthropometric characteristics of subjects

Characteristics	n (%)	Mean±SD
Gender		
Male Female	56 (49.6) 57 (50.4)	
Age (years)		
<60 ≥60	77 (68.1) 36 (31.9)	54.05±10.30
Ethnicity		
Malay Chinese Indian Others	52 (46.0) 32 (28.3) 26 (23.0) 3 (2.7)	
Marital status		
Single Married Widowed	5 (4.4) 95 (84.1) 13 (11.5)	
Educational level		
None/Primary Secondary Tertiary	37 (32.7) 56 (49.6) 20 (17.7)	9.23±4.38
Working status		
Working Not working	48 (42.5) 65 (57.5)	
Personal income (MYR)*		
Low (<500) Medium (500-1,999) High (≥2,000)	42 (37.2) 33 (29.2) 38 (33.6)	
Household income (MYR)*		
Low (<3,000) Medium (3,000-4,999) High (≥5,000)	53 (46.9) 29 (25.7) 31 (27.4)	
Duration of diabetes (years)		
<5 5-9.9 ≥10	33 (29.2) 21 (18.6) 59 (52.2)	11.25±9.05
Type of medication		
None Pills only Insulin only Pills and insulin	0 (0.0) 48 (42.5) 2 (1.8) 63 (55.8)	
Weight (kg)		76.35±17.70
Height (cm)		159.52±9.40
Body mass index (kg/m^2^)		
Underweight Normal range Overweight Pre-obese Obese Class I Obese Class II	0 (0.0) 7 (6.2) 33 (29.2) 48 (42.5) 14 (12.4) 11 (9.7)	29.82±5.45
Waist-circumference (cm)		98.49±13.34
Normal At risk	14 (12.4) 99 (87.6)	
Hip-circumference (cm)		104.26±10.69
Waist-to-hip ratio		
Normal At risk	29 (25.7) 84 (74.3)	0.94±0.07

*1.00 MYR was equivalent to US$ 0.32 at the time of study; SD=Standard deviation; MYR=Malaysian Ringgit

Table 4 displays the strength of the associations between FGS and characteristics of the subjects in simple and multiple linear regression models. Among the eight variables that were selected (p<0.25) to be entered into the final model, only five were found to predict significantly the number of food group intake as measured by FGS [F (6,106)=4.924, p<0.0001]. Educational level was positively associated with FGS, with every one year increase in education contributing to 0.05 unit increase in FGS (p=0.005). Among individuals with T2DM, those who were not working, had high personal income, on insulin regimen, and of normal weight were found to have more diversified diet than their counterparts, ranging from 0.3-0.7 food group more each day (p<0.05).

On the other hand, only three out of eight variables were found to predict significantly the presence of achieving the minimum recommended number of servings of various food groups as measured by SS [F (4,108)=7.520, p<0.0001] (Table 5). Educational level was positively associated with SS, with each year increase in education contributing to 0.29 unit increase in SS (p<0.0001). Subjects who were of normal weight were found to have approximately 4 units higher in SS than their overweight, pre-obese or obese counterparts (p<0.05). On the other hand, WHR was positively associated with SS, with one unit increase in WHR contributing to 9.58 units increase in SS (p=0.025). The final models explained about 21.8% of the variance in the scores for FGS and SS. The Durbin-Watson coefficients were found to be approaching 2, indicating the data were independent, and there was no autocorrelation in the sample.

## DISCUSSION

This study demonstrates that a high proportion of subjects with T2DM failed to adhere to the national dietary guidelines. Nearly two-thirds of the study subjects with T2DM did not consume all five major food groups daily. Only about half of the subjects fulfilled the minimum recommended number of servings of each food group. In contrast to earlier findings from studies conducted among free-living community-based Malaysians, which reported that only dairy consumption was below the suggested intake (23), our findings were consistent with other studies done in European countries among individuals with diabetes (24,25). This suggests that, although diet is acknowledged as a very critical component in the overall treatment for T2DM, poor adherence is highly expected.

A possible explanation for non-adherence to national dietary guideline might be that individuals with T2DM were looking for ‘diabetic diet’. In this study, dairy and fruits are the least-frequently consumed food groups. Some respondents reported that they tried to avoid these foods because they perceived dairy is potentially fattening (16.7%) and fruit is ‘too sweet’ (19.0%) for people with diabetes. In fact, nutritional recommendations for individuals with T2DM resemble the ‘healthy pattern of diet’ for the general population as shown in the national dietary guidelines. A well-balanced meal is equally important for preventing and controlling diabetes for both individuals with or without diabetes (9,26). A healthy and well-balanced diet should meet the national dietary guidelines that encourage people to eat a variety of foods that include carbohydrate from fruits, vegetables, whole grains, legumes, and low-fat milk; to monitor portion-sizes; to eat a variety of fibre-containing foods; to consume protein in moderation; and to reduce intakes of energy, saturated and trans-fatty acids, cholesterol as well as sodium (9). Studies have shown that consuming a diet with fruits, vegetables, whole grains, low-fat dairy, lean meats, and alternatives was not only associated with a lower risk of all-cause mortality (27), it also had considerable merit for improving health outcome (28).

**Table 3.** Distribution of subjects according to dietary diversity score

Food group	Dietary diversity score			
	Food group Score*		Serving score†	
	n (%)	Mean±SD	n (%)	Mean±SD
Grain Vegetables Meat Fruits Dairy Summary score Perfect scoring§	113 (100.0)‡ 108 (95.6)‡ 110 (97.3)‡ 86 (76.1)‡ 49 (43.4)‡ 39 (34.5)	1.00±0.00 0.96±0.21 0.97±0.16 0.76±0.43 0.43±0.50 4.12±0.79	61 (54.0)¶ 58 (51.3)¶ 44 (38.9)¶ 33 (29.2)¶ 12 (10.6)¶ 2 (1.8)	3.42±0.78 3.06±1.11 2.98±0.98 2.20±1.48 1.09±1.37 12.75±3.50

*Food Group Score (FGS) counts the number of food groups consumed daily from a total of five groups, namely grains, fruits, vegetables, meat, and dairy. One point was given for each food group;

^†^Serving Score (SS) evaluates the presence of achieving the minimum recommended number of servings for the various food groups—four servings daily from grains group and two servings for each of the remaining groups. One point was given for each serving of grains and two points for other food groups;

^‡^Proportion of subjects consuming the food group daily;

^¶^Proportion of subjects achieving the recommended number of servings of the food group daily;

§Perfect score for Food Group Score was 5 and, for Serving Score, it was 20

Dairy products are frequently perceived as fattening and should be avoided to prevent obesity. In fact, a review done by Zemel (29) found evidence for the presence of an anti-obesity effect of dairy foods. Dairy foods were also found to be inversely associated with the development of intra-abdominal adipose tissues (30). Besides the anti-obesity effects, dairy intake was found to be inversely associated with the development of abnormal glucose homeostasis, elevated blood pressure, and dyslipidaemia among overweight adults (31). All these components are particularly crucial for preventing diabetes complications among individuals with T2DM (9).

The present study shows a direct association between level of education and DDS. This finding was consistent with other studies (32-34) which found that lower education was associated with less diversified and poor diet quality. These results may be explained by the fact that certain literacy level is required to comprehend the available health information. Less-educated subjects may find it hard to make use of written materials, like newspaper articles and leaflets, to gain nutritional and health-related knowledge (35). Understanding the information regarding diet-disease links can be complex and challenging for these vulnerable subjects and, hence, limit their ability to implement the nutritional and health-related knowledge in daily life (35). Norimah *et al*. (36) demonstrated that the key words and key messages in the newly-updated MDG are poorly understood by less-educated Malaysians, especially those with only primary education. Although the importance of taking well-balanced diet with various food groups to prevent diet-related chronic diseases has existed for more than a decade in Malaysia (37), such messages fail to reach their target audience, especially to those with lower educational level. As higher prevalence of T2DM was documented amongst lower-educated subjects, there is an urgent need to promote nutritional awareness with messages that are tailor-made for this group. In this context, the USDA's newest visual tool—MyPlate—might serve as one of the options as many new users perceived it as simple, visually appealing, and could be personalized to fit their diet (38).

**Table 4.** Associations between Food Group Score (FGS) and characteristics of sociodemographic, diabetes history, and anthropometric measures in simple and multiple regression models

Characteristics	Simple regression^†^		Multiple regression^‡^	
	β (95% CI)	p value	β (95% CI)	p value
Sociodemographic characteristics				
Gender (male vs female) Age (years) Ethnicity (Chinese vs Malay) Ethnicity (Indian vs Malay) Marital status (single/widowed vs married) Education (years) Working status (non-working vs working) Personal income (medium vs low) Personal income (high vs low) Household income (medium vs low) Household income (high vs low)	0.073 (-0.22, 0.37) -0.01 (-0.02, 0.01) -0.22 (-0.58, −0.13) -0.20 (-0.38, 0.34) -0.15 (-0.55, 0.26) 0.04 (0.01, 0.07) 0.22 (-0.08, 0.51) -0.02 (-0.38, 0.35) 0.24 (-0.11, 0.59) 0.35 (-0.00, 0.71) 0.40 (0.05, 0.75)	0.626 0.449 **0.212** 0.914 0.472 **0.024** **0.154** 0.925 **0.174** **0.053** **0.025**	0.05 (0.02, 0.09) 0.52 (0.18, 0.86) 0.37 (0.00, 0.73)	0.005** 0.003** 0.048*
Diabetes history				
Duration of diabetes (years) Type of treatment (with insulin vs no insulin)	0.01 (-0.01, 0.03) 0.29 (-0.01, 0.58)	**0.156** **0.056**	0.36 (0.08, 0.63)	0.012*
Anthropometric measures				
BMI (overweight vs normal range) BMI (pre-obese/obese vs normal range) Waist-circumference (cm) Waist-to-hip ratio	-0.62 (-1.27, 0.02) -0.63 (-1.25, −0.02) -0.00 (-0.01, 0.10) 1.08 (-0.95, 3.10)	**0.059** **0.044** 0.897 0.294	-0.68 (-1.30, −0.07) -0.67 (-1.25, −0.08)	0.028* 0.026*

Variables that had a p<0.25 in the simple linear regression models are shown in **bold** and were chosen for backward stepwise multiple linear regression analysis.

^†^Simple linear regression: association is significant at *p<0.004 (Bonferroni adjustment);

^‡^Backward stepwise multiple linear regression: association is significant at *p<0.05, **p<0.01, R=0.467, R^2^=0.218, Adjusted R^2^=0.174, F (6,106)=4.924, p<0.0001, Durbin-Watson=1.943; β=Coefficient; BMI=Body mass index; CI=Confidence interval

**Table 5.** Associations between Serving Score (SS) and sociodemographics, diabetes history, and anthropometric measures in simple and multiple regression models

Characteristics	Simple regression†		Multiple regression‡	
	β (95% CI)	p value	β (95% CI)	p value
Sociodemographic characteristics				
Gender (male vs female) Age (years) Ethnicity (Chinese vs Malay) Ethnicity (Indian vs Malay) Marital status (single/widowed vs married) Education (years) Working status (non-working vs working) Personal income (medium vs low) Personal income (high vs low) Household income (medium vs low) Household income (high vs low)	1.61 (0.33, 2.88) -0.07 (-0.13, −0.01) -1.29 (-2.84, 0.26) -1.34 (-2.94, 0.25) -1.03 (-2.81, 0.76) 0.28 (0.14, 0.42) -0.56 (-1.88, 0.77) 0.49 (-1.07, 2.06) 2.24 (0.73, 3.74) 1.14 (-0.44, 2.72) 1.81 (0.27, 3.36)	**0.014** **0.031** **0.101** **0.099** 0.256 0.000* 0.406 0.535 **0.004** **0.155** **0.022**	0.29 (0.15, 0.43)	0.000***
Diabetes history				
Duration of diabetes (years) Type of treatment (with insulin vs no insulin)	-0.01 (-0.08, 0.06) -0.18 (-1.50, 1.15)	0.801 0.792		
Anthropometric measures				
BMI (overweight vs normal range) BMI (pre-obese/obese vs normal range) Waist-circumference (cm) Waist-to-hip ratio	-2.54 (-5.41, 0.33) -2.55 (-5.28, 0.18) 0.03 (-0.03, 0.07) 10.06 (1.26, 18.86)	**0.083** **0.066** 0.324 **0.025**	-3.85 (-6.50, −1.20) -3.87 (-6.41, −1.33) 9.58 (1.24, 17.92)	0.005** 0.003** 0.025*

Variables that had a p<0.25 in the simple linear regression models are shown in **bold** and were chosen for backward stepwise multiple linear regression analysis.

^†^Simple linear regression, association is significant at *p<0.004 (Bonferroni adjustment);

^‡^Backward stepwise multiple linear regression, association is significant at *p<0.05, **p<0.01, ***p<0.001, R=0.467, R^2^=0.218, Adjusted R^2^=0.189, F (4,108)=7.520, p<0.0001, Durbin-Watson=2.164; β=Coefficient; BMI=Body mass index; CI=Confidence interval

Our data are in agreement with the findings reported in a review paper by Darmon and Drewnowski (39) that showed better-quality diets are mainly consumed by better-educated and more-affluent people and suggested that the observed socioeconomic status gradient in diet quality may be mediated by prices of food and costs of diet. Prices of food could be a very important determinant of food choices and diet quality as low-income group spend a relatively higher proportion of their income on food than higher-income group does (40). Individuals with low educational level and limited income were more likely to perceive food price as very important, which could further influence their food-purchasing decisions and, consequently, impact on their diet quality (41). Bowman (41) found that those who perceived food price as very important were more likely to eat a low amount of relatively high-price foods and consume more energy-dense poor-nutrient diets.

Our data reveal that working diabetics are more likely to eat a less diversified diet. This finding may be particularly true as working subjects may have more workloads and working demands than do their non-working counterparts. They may be more likely to experience feelings of time scarcity and lack of energy which may further alter their food choices, such as eating out, eating on the run, eating junk foods, or skipping meals. Lin *et al*. (42) found that eating out or consuming foods away from home was associated with poorer nutritional quality, which typically contained more in fat and saturated fat and less in calcium, fibre and iron compared to home-made foods. These were probably true in the present study, where the number of food groups consumed was found to be strongly and positively associated with nutritional quality (11). Devine *et al*. (43) suggested that many workers may have sufficient information about healthy dietary choices but, due to work constraint, they perceive that they cannot put food choice ideals into practice. As a result, convenience seems to be the most important factor above personal health for working subjects when dealing with food choices in lunch (44). These may explain why this group of people had poorer diet quality, and nutrition intervention should include specific strategies to addressing the problems.

Type of medication is also found to predict the dietary quality significantly in the present study. Subjects with T2DM, who were on insulin regimen, tended to eat more food groups compared to their counterparts not on insulin regimen. The possible explanations for the differences are that those on insulin regimen might be more likely to feel better and to have less symptoms of dizziness, depression, fatigue, thirst and dry mouth, polyuria, and nocturia than their non-insulin counterparts (45,46). This better general wellbeing may lead to better appetite and feeling safe to include various food groups into their diet. In contrast, non-insulin group may be more cautious to take certain type of food groups which they perceived as forbidden, especially during poorly-controlled conditions; therefore, consuming less number of food groups than their insulin counterparts. To the best of our knowledge, there are inadequate studies on diet quality and type of medication used among individuals with T2DM. Diet therapy is one of the most important interventions for diabetes for those newly-diagnosed with T2DM. Thus, it is important for healthcare providers to reinforce the healthy eating concept for better diabetes management.

It is somewhat surprising that our data show contradictory results between the two anthropometric measures, namely BMI and WHR in predicting DDS. BMI seemed to be negatively associated while WHR was positively associated with DDS. Subjects with normal weight consumed significantly higher number of food groups and higher number of servings in all food groups, which indicated a better diet quality. On the other hand, subjects with higher WHR consumed significantly higher number of servings in all food groups and, in turn, had better diet quality. Inconsistent findings were found in the literature regarding the association between diet quality, obesity, and abdominal adiposity. Better diet quality or specifically more diversified and varied diets were often found to be associated with higher energy intake and, hence, obesity (47-50). However, such association may be affected by the nutrient contents of the diet or the distribution of the energy across various food groups. In a study by Azadbakht *et al*. (51), although subjects with higher DDS had significantly higher energy intake, such increase in energy intakes was attributed by the increased intake of healthy food groups and low-energy food groups and, hence, were inversely related to obesity and abdominal adiposity. This double-edged diversity may, hence, make the potential associations between diet quality and nutritional status difficult to reveal (52).

### Limitations

This study has several limitations. First, the cross-sectional design did not allow us to rule out the direction of the associations, especially the association between diet quality and nutritional status. Second, the study subjects were individuals with T2DM, who had undergone routine check-ups and, hence, dietary changes among them were very likely. To address this shortcoming, food frequency questionnaire, instead of 24-hour dietary recall, was used for determining dietary diversity based on their usual dietary intake. Third, like other DDS studies (13,14,22), the definition of FGS and SS were limited to setting the minimum levels but not the upper limits, which restricted us from capturing those who had over-consumed. It is noteworthy that, although DDS is a relatively simple index that does not require any quantitative estimation of serving-size, this index was a positive predictor of dietary biomarkers (53) and nutrient adequacy (11,12), a negative predictor of CVD risk (13), and metabolic syndrome (14). However, a review of DDS suggested that this index might be improved by applying a minimum portion-size (54). As little as 10 g of cutoff was found to improve the sensitivity and specificity of the index and improve the ability to predict nutrient adequacy (55). Acknowledgeing the limitations of this study, the present study, nevertheless, is pertinent to other researchers and individuals with T2DM as research on diet quality is scarce. Diet is one of the most important treatments for individuals with T2DM where proper dietary intake could help them get rid of or to delay the development of diabetes complications.

### Conclusions

The findings of this study demonstrate that high proportion of individuals with T2DM failed to adhere to the national dietary guidelines. Those with lower education, working, had low personal income, currently not on insulin regimen, being overweight or obese, and those with lower WHR were found to have a significantly poorer dietary diversity. The importance of taking a well-balanced diet in accordance with the national dietary guidelines should be emphasized among individuals with T2DM, especially those with lower education through a simple and easy-to-understand approach.

## ACKNOWLEDGEMENTS

This research received no specific grant from any funding agency in the public, commercial or not-for-profit sectors. The authors are grateful to SW Food International (M) Pte Ltd. and Fonterra Brands (Malaysia) Pte Ltd. for their kind support as well as to all the participants from Serdang Hospital and the University Health Clinic of UPM for their enthusiastic cooperation.

**Conflict of interest:** The authors have no conflict of interest.
